# Poly[[[silver(I)-μ-1,4-bis­[(imidazol-1-yl)meth­yl]benzene-κ^2^ *N* ^3^:*N* ^3′^-silver(I)-μ-1,4-bis­[(imidazol-1-yl)meth­yl]benzene-κ^2^ *N* ^3^:*N* ^3′^] 4,4′-diazenediyldibenzoate] dihydrate]

**DOI:** 10.1107/S1600536811008816

**Published:** 2011-03-23

**Authors:** Qian Qiao, Mei Tian, Da-Yong Liu, Shu-Jiang Wang

**Affiliations:** a College of Chemical Engineering, Changchun University of Technology, Changchun 130012, People’s Republic of China

## Abstract

In the title compound, [Ag_2_(C_14_H_14_N_4_)_2_](C_14_H_8_N_2_O_4_)·2H_2_O, each of the two unique Ag^+^ ions is two-coordinated by two N atoms from two different 1,4-bis­[(imidazol-1-yl)meth­yl]benzene ligands in an almost linear fashion [N—Ag—N = 170.34 (10) and 160.25 (10)°]. The 4,4′-diazenediyldibenzoate anions do not coordinate to Ag. O—H⋯O hydrogen bonds stabilize the crystal structure.

## Related literature

For a related structure, see: Xu *et al.* (2005 ▶). For applications of coordination polymers, see: Chen *et al.* (2008 ▶).
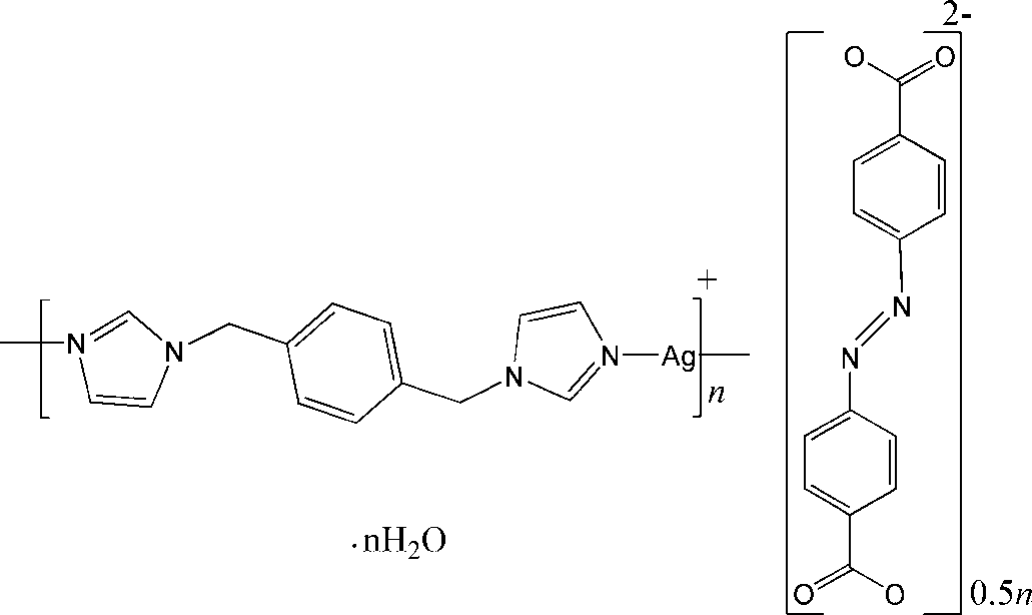


## Experimental

### 

#### Crystal data

[Ag_2_(C_14_H_14_N_4_)_2_](C_14_H_8_N_2_O_4_)·2H_2_O
*M* *_r_* = 996.58
Triclinic, 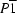

*a* = 9.4944 (6) Å
*b* = 9.7810 (7) Å
*c* = 24.4049 (9) Å
α = 84.111 (4)°
β = 88.765 (4)°
γ = 63.245 (7)°
*V* = 2012.3 (2) Å^3^
*Z* = 2
Mo *K*α radiation
μ = 1.04 mm^−1^
*T* = 293 K
0.30 × 0.24 × 0.21 mm

#### Data collection

Oxford Diffraction Gemini R Ultra diffractometer
Absorption correction: multi-scan (*CrysAlis RED*; Oxford Diffraction, 2006 ▶) *T*_min_ = 0.42, *T*_max_ = 0.74
13525 measured reflections
8145 independent reflections
5125 reflections with *I* > 2σ(*I*)
*R*_int_ = 0.018

#### Refinement

*R*[*F* ^2^ > 2σ(*F* ^2^)] = 0.040
*wR*(*F* ^2^) = 0.102
*S* = 1.02
8145 reflections
541 parameters
6 restraints
H-atom parameters constrained
Δρ_max_ = 1.38 e Å^−3^
Δρ_min_ = −1.04 e Å^−3^

### 

Data collection: *CrysAlis CCD* (Oxford Diffraction, 2006 ▶); cell refinement: *CrysAlis CCD*; data reduction: *CrysAlis RED* (Oxford Diffraction, 2006 ▶); program(s) used to solve structure: *SHELXS97* (Sheldrick, 2008 ▶); program(s) used to refine structure: *SHELXL97* (Sheldrick, 2008 ▶); molecular graphics: *SHELXTL* (Sheldrick, 2008 ▶); software used to prepare material for publication: *SHELXTL*.

## Supplementary Material

Crystal structure: contains datablocks global, I. DOI: 10.1107/S1600536811008816/bt5474sup1.cif

Structure factors: contains datablocks I. DOI: 10.1107/S1600536811008816/bt5474Isup2.hkl

Additional supplementary materials:  crystallographic information; 3D view; checkCIF report

## Figures and Tables

**Table 1:** Hydrogen-bond geometry (Å, °)

*D*—H⋯*A*	*D*—H	H⋯*A*	*D*⋯*A*	*D*—H⋯*A*
O1*W*—H*W*12⋯O2*W*^i^	0.85	2.11	2.879 (5)	151
O1*W*—H*W*11⋯O3^i^	0.85	2.02	2.828 (4)	159
O2*W*—H*W*21⋯O4	0.85	1.93	2.758 (4)	163
O2*W*—H*W*22⋯O4^ii^	0.85	2.18	2.909 (6)	144

Symmetry codes: (i) 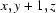; (ii) 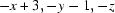.

**Fig. 1.**
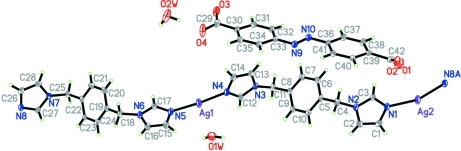
The structure of the title compound, showing the atomic numbering scheme. Displacement ellipsoids are drawn at the 30% probability level. Symmetry code: (i) x-2, 1+y, 1+z.

## supplementary crystallographic information

### Comment

Coordination polymers are of considerable interest because of their potential application in the areas of catalysis, separation, sorption, sensors, and in electronic and magnetic devices (Chen *et al.*, 2008). So far, the rigid rod-like spacer is well known in the construction of coordination polymers. However, the coordination polymers with the flexible N-donor ligands such as 1,4-bis(imidazole-1-ylmethyl)benzene (1,4-bix) and dicatrboxylic acids such as 4,4'-diazenediyldibenzoic acid (H_2_L) have not been well explored (Xu *et al.*, 2005). Here, 1,4-bix assembles with silver and H_2_L to yield a coordination polymer [Ag_2_(1,4-bix)]^.^L^.^2H_2_O, (I).

As shown in Fig. 1, there are two Ag(I) atoms, one 1,4-bix ligand, one L anion, and two free water molecules in the asymmetric unit. Each Ag atom ( Ag1 or Ag2) is two-coordinated by two nitrogen atoms from two different 1,4-bix ligands in a linear sphere. The Ag atoms are bridged by the 1,4-bix ligands to generate a chain structure. Notably, the L anion did not coordinate to the Ag center, but acted as a counter-anion. The O-H···O hydrogen bonds further stabilize the chain structure (Table 1).

### Experimental

A mixture of AgNO_3_^.^2H_2_O (0.5 mmol), H_2_L (0.5 mmol), 1,4-bix (0.5 mmol), and H_2_O (12 ml) was adjusted to pH = 5-6 by addition of aqueous NaOH solution, and heated at 140 ^o^C for 3 days. After the mixture was slowly cooled to room temperature, crystals of (I) were yielded (27% yield).

### Refinement

All H atoms on C atoms were positioned geometrically (C—H = 0.93 Å) and refined as riding, with *U*_iso_(H)=1.2*U*_eq_(C). H atoms bonded to water molecules were located in difference Fourier maps and refined isotropically with distance restraints of O—H = 0.85±0.01 and H···H = 1.35 ±0.01 Å with U_iso_ = 1.5U_eq_(O).

### Crystal data

**Table:**

[Ag_2_(C_14_H_14_N_4_)_2_](C_14_H_8_N_2_O_4_)·2H_2_O	*Z* = 2
*M**_r_* = 996.58	*F*(000) = 1008
Triclinic, *P*1	*D*_x_ = 1.645 Mg m^−^^3^
Hall symbol: -P 1	Mo *K*α radiation, λ = 0.71073 Å
*a* = 9.4944 (6) Å	Cell parameters from 8145 reflections
*b* = 9.7810 (7) Å	θ = 4.2–26.4°
*c* = 24.4049 (9) Å	µ = 1.04 mm^−^^1^
α = 84.111 (4)°	*T* = 293 K
β = 88.765 (4)°	Block, pale yellow
γ = 63.245 (7)°	0.30 × 0.24 × 0.21 mm
*V* = 2012.3 (2) Å^3^	

### Data collection

**Table:**

Oxford Diffraction Gemini R Ultra diffractometer	8145 independent reflections
Radiation source: fine-focus sealed tube	5125 reflections with *I* > 2σ(*I*)
graphite	*R*_int_ = 0.018
Detector resolution: 10.0 pixels mm^-1^	θ_max_ = 26.4°, θ_min_ = 4.2°
ω scans	*h* = −11→11
Absorption correction: multi-scan (*CrysAlis RED*; Oxford Diffraction, 2006)	*k* = −12→12
*T*_min_ = 0.42, *T*_max_ = 0.74	*l* = −24→30
13525 measured reflections	

### Refinement

**Table:**

Refinement on *F*^2^	Primary atom site location: structure-invariant direct methods
Least-squares matrix: full	Secondary atom site location: difference Fourier map
*R*[*F*^2^ > 2σ(*F*^2^)] = 0.040	Hydrogen site location: inferred from neighbouring sites
*wR*(*F*^2^) = 0.102	H-atom parameters constrained
*S* = 1.01	*w* = 1/[σ^2^(*F*_o_^2^) + (0.0528*P*)^2^] where *P* = (*F*_o_^2^ + 2*F*_c_^2^)/3
8145 reflections	(Δ/σ)_max_ = 0.001
541 parameters	Δρ_max_ = 1.38 e Å^−^^3^
6 restraints	Δρ_min_ = −1.04 e Å^−^^3^

### Special details

**Table:**

Geometry. All esds (except the esd in the dihedral angle between two l.s. planes) are estimated using the full covariance matrix. The cell esds are taken into account individually in the estimation of esds in distances, angles and torsion angles; correlations between esds in cell parameters are only used when they are defined by crystal symmetry. An approximate (isotropic) treatment of cell esds is used for estimating esds involving l.s. planes.
Refinement. Refinement of F^2^ against ALL reflections. The weighted R-factor wR and goodness of fit S are based on F^2^, conventional R-factors R are based on F, with F set to zero for negative F^2^. The threshold expression of F^2^ > 2sigma(F^2^) is used only for calculating R-factors(gt) etc. and is not relevant to the choice of reflections for refinement. R-factors based on F^2^ are statistically about twice as large as those based on F, and R- factors based on ALL data will be even larger.

### Fractional atomic coordinates and isotropic or equivalent isotropic displacement parameters (Å^2^)

**Table:**

	*x*	*y*	*z*	*U*_iso_*/*U*_eq_	
C1	0.4668 (4)	0.6968 (4)	0.36130 (15)	0.0563 (9)	
H1	0.4077	0.8025	0.3622	0.068*	
C2	0.5853 (4)	0.6290 (4)	0.32737 (14)	0.0556 (9)	
H2	0.6228	0.6782	0.3006	0.067*	
C3	0.5543 (3)	0.4537 (4)	0.38025 (12)	0.0423 (7)	
H3	0.5684	0.3582	0.3967	0.051*	
C4	0.7804 (4)	0.3517 (4)	0.31781 (13)	0.0587 (10)	
H4A	0.8024	0.2545	0.3390	0.070*	
H4B	0.8700	0.3721	0.3228	0.070*	
C5	0.7643 (3)	0.3364 (4)	0.25806 (12)	0.0427 (7)	
C6	0.7031 (4)	0.2437 (4)	0.24143 (14)	0.0556 (9)	
H6	0.6644	0.1941	0.2676	0.067*	
C7	0.6983 (4)	0.2230 (4)	0.18644 (14)	0.0574 (9)	
H7	0.6568	0.1593	0.1761	0.069*	
C8	0.7542 (3)	0.2957 (4)	0.14689 (12)	0.0414 (7)	
C9	0.8099 (4)	0.3932 (4)	0.16302 (13)	0.0482 (8)	
H9	0.8429	0.4473	0.1366	0.058*	
C10	0.8176 (4)	0.4119 (4)	0.21810 (13)	0.0511 (8)	
H10	0.8590	0.4758	0.2284	0.061*	
C11	0.7589 (3)	0.2668 (4)	0.08728 (13)	0.0521 (8)	
H11A	0.6876	0.2234	0.0809	0.062*	
H11B	0.7233	0.3638	0.0642	0.062*	
C12	1.0059 (4)	0.1906 (4)	0.03344 (13)	0.0504 (8)	
H12	0.9732	0.2842	0.0118	0.060*	
C13	1.0080 (4)	0.0133 (4)	0.09413 (14)	0.0551 (9)	
H13	0.9794	−0.0401	0.1223	0.066*	
C14	1.1443 (4)	−0.0405 (4)	0.06767 (14)	0.0553 (9)	
H14	1.2268	−0.1392	0.0743	0.066*	
C15	1.4332 (4)	0.1450 (5)	−0.14000 (15)	0.0609 (9)	
H15	1.3515	0.2445	−0.1411	0.073*	
C16	1.5447 (4)	0.0934 (5)	−0.17812 (14)	0.0605 (10)	
H16	1.5539	0.1493	−0.2100	0.073*	
C17	1.5860 (4)	−0.0903 (4)	−0.11316 (13)	0.0504 (8)	
H17	1.6319	−0.1859	−0.0924	0.061*	
C18	1.7908 (4)	−0.1566 (5)	−0.18476 (13)	0.0586 (9)	
H18A	1.8364	−0.2558	−0.1628	0.070*	
H18B	1.8631	−0.1120	−0.1825	0.070*	
C19	1.7766 (3)	−0.1816 (4)	−0.24384 (12)	0.0422 (7)	
C20	1.7362 (4)	−0.2936 (4)	−0.25662 (13)	0.0580 (9)	
H20	1.7087	−0.3481	−0.2286	0.070*	
C21	1.7358 (4)	−0.3263 (4)	−0.31030 (14)	0.0569 (9)	
H21	1.7089	−0.4030	−0.3178	0.068*	
C22	1.7748 (3)	−0.2467 (4)	−0.35278 (11)	0.0390 (7)	
C23	1.8106 (3)	−0.1309 (4)	−0.34061 (12)	0.0444 (7)	
H23	1.8338	−0.0736	−0.3689	0.053*	
C24	1.8123 (4)	−0.0996 (4)	−0.28692 (13)	0.0478 (8)	
H24	1.8378	−0.0219	−0.2794	0.057*	
C25	1.7813 (3)	−0.2857 (4)	−0.41137 (13)	0.0505 (8)	
H25A	1.7261	−0.3475	−0.4142	0.061*	
H25B	1.7272	−0.1913	−0.4356	0.061*	
C26	2.1755 (4)	−0.5495 (4)	−0.44614 (14)	0.0535 (9)	
H26	2.2632	−0.6446	−0.4467	0.064*	
C27	2.0211 (3)	−0.3084 (4)	−0.46087 (12)	0.0421 (7)	
H27	1.9806	−0.2044	−0.4733	0.050*	
C28	2.0419 (4)	−0.5236 (4)	−0.41991 (14)	0.0531 (8)	
H28	2.0203	−0.5966	−0.3990	0.064*	
C29	1.2536 (4)	−0.4939 (4)	0.08366 (14)	0.0568 (9)	
C30	1.1939 (4)	−0.4119 (4)	0.13444 (12)	0.0427 (7)	
C31	1.0345 (4)	−0.3561 (4)	0.14751 (13)	0.0478 (8)	
H31	0.9656	−0.3669	0.1238	0.057*	
C32	0.9781 (4)	−0.2866 (4)	0.19416 (13)	0.0481 (8)	
H32	0.8713	−0.2482	0.2015	0.058*	
C33	1.0808 (4)	−0.2725 (4)	0.23113 (12)	0.0426 (7)	
C34	1.2384 (4)	−0.3263 (4)	0.21846 (13)	0.0498 (8)	
H34	1.3074	−0.3169	0.2425	0.060*	
C35	1.2940 (4)	−0.3936 (4)	0.17063 (13)	0.0492 (8)	
H35	1.3998	−0.4272	0.1624	0.059*	
C36	0.8488 (3)	−0.1055 (3)	0.34361 (12)	0.0383 (7)	
C37	0.6912 (4)	−0.0543 (4)	0.35628 (13)	0.0471 (8)	
H37	0.6233	−0.0666	0.3326	0.057*	
C38	0.6354 (3)	0.0141 (4)	0.40339 (13)	0.0469 (8)	
H38	0.5293	0.0482	0.4112	0.056*	
C39	0.7335 (3)	0.0341 (3)	0.44001 (11)	0.0372 (7)	
C40	0.8927 (3)	−0.0219 (3)	0.42703 (12)	0.0388 (7)	
H40	0.9614	−0.0129	0.4513	0.047*	
C41	0.9501 (3)	−0.0888 (3)	0.38049 (12)	0.0399 (7)	
H41	1.0564	−0.1237	0.3729	0.048*	
C42	0.6712 (4)	0.1177 (4)	0.49038 (12)	0.0425 (7)	
N1	0.4465 (3)	0.5863 (3)	0.39436 (11)	0.0491 (7)	
N2	0.6407 (3)	0.4737 (3)	0.33959 (9)	0.0410 (6)	
N3	0.9191 (3)	0.1608 (3)	0.07206 (10)	0.0421 (6)	
N4	1.1436 (3)	0.0707 (3)	0.02991 (11)	0.0522 (7)	
N5	1.4586 (3)	0.0284 (4)	−0.09930 (11)	0.0538 (7)	
N6	1.6413 (3)	−0.0567 (3)	−0.16084 (10)	0.0457 (6)	
N7	1.9436 (3)	−0.3701 (3)	−0.42960 (9)	0.0371 (6)	
N8	2.1618 (3)	−0.4131 (3)	−0.47186 (11)	0.0487 (7)	
N9	1.0353 (3)	−0.2065 (3)	0.28132 (11)	0.0480 (6)	
N10	0.8960 (3)	−0.1731 (3)	0.29367 (10)	0.0462 (6)	
O1	0.5368 (3)	0.2259 (3)	0.48619 (10)	0.0694 (7)	
O2	0.7631 (3)	0.0773 (3)	0.53134 (9)	0.0601 (6)	
O3	1.1669 (3)	−0.5339 (4)	0.06016 (11)	0.0797 (8)	
O4	1.3891 (3)	−0.5200 (5)	0.06974 (12)	0.1081 (12)	
Ag1	1.31507 (3)	0.04951 (4)	−0.029913 (12)	0.07450 (13)	
Ag2	0.31551 (3)	0.61261 (4)	0.467482 (11)	0.06951 (13)	
O1W	1.1940 (4)	0.3750 (4)	−0.04740 (11)	0.1012 (11)	
HW11	1.1640	0.4002	−0.0154	0.152*	
HW12	1.2938	0.3250	−0.0444	0.152*	
O2W	1.5129 (4)	−0.7010 (4)	−0.01539 (14)	0.1170 (13)	
HW21	1.4571	−0.6378	0.0069	0.176*	
HW22	1.5218	−0.6454	−0.0430	0.176*	

### Atomic displacement parameters (Å^2^)

**Table:**

	*U*^11^	*U*^22^	*U*^33^	*U*^12^	*U*^13^	*U*^23^
C1	0.056 (2)	0.035 (2)	0.067 (2)	−0.0095 (16)	0.0096 (17)	−0.0138 (17)
C2	0.059 (2)	0.045 (2)	0.055 (2)	−0.0180 (17)	0.0104 (17)	−0.0005 (17)
C3	0.0488 (18)	0.044 (2)	0.0353 (16)	−0.0203 (15)	0.0054 (14)	−0.0105 (14)
C4	0.0437 (18)	0.061 (2)	0.0429 (18)	0.0025 (16)	0.0034 (15)	−0.0107 (16)
C5	0.0359 (15)	0.044 (2)	0.0364 (16)	−0.0069 (14)	0.0091 (13)	−0.0113 (14)
C6	0.063 (2)	0.059 (2)	0.047 (2)	−0.0310 (19)	0.0211 (16)	−0.0049 (17)
C7	0.065 (2)	0.064 (3)	0.057 (2)	−0.039 (2)	0.0157 (17)	−0.0183 (18)
C8	0.0323 (15)	0.0435 (19)	0.0416 (17)	−0.0099 (14)	0.0073 (13)	−0.0112 (14)
C9	0.0570 (19)	0.047 (2)	0.0417 (18)	−0.0242 (17)	0.0168 (14)	−0.0076 (15)
C10	0.055 (2)	0.053 (2)	0.052 (2)	−0.0281 (17)	0.0137 (16)	−0.0189 (16)
C11	0.0397 (17)	0.066 (2)	0.0403 (17)	−0.0130 (16)	0.0051 (14)	−0.0167 (16)
C12	0.060 (2)	0.044 (2)	0.0385 (18)	−0.0164 (17)	0.0111 (15)	−0.0044 (15)
C13	0.070 (2)	0.042 (2)	0.050 (2)	−0.0228 (18)	0.0162 (17)	−0.0037 (16)
C14	0.060 (2)	0.040 (2)	0.052 (2)	−0.0091 (17)	0.0122 (17)	−0.0110 (17)
C15	0.051 (2)	0.060 (3)	0.058 (2)	−0.0127 (18)	0.0076 (17)	−0.0095 (19)
C16	0.060 (2)	0.062 (3)	0.047 (2)	−0.018 (2)	0.0122 (17)	−0.0001 (18)
C17	0.061 (2)	0.053 (2)	0.0388 (18)	−0.0260 (18)	0.0083 (15)	−0.0118 (15)
C18	0.0446 (18)	0.079 (3)	0.0432 (19)	−0.0175 (18)	0.0087 (15)	−0.0206 (18)
C19	0.0333 (15)	0.051 (2)	0.0381 (17)	−0.0142 (14)	0.0067 (12)	−0.0112 (14)
C20	0.075 (2)	0.069 (3)	0.0405 (19)	−0.043 (2)	0.0129 (17)	−0.0024 (17)
C21	0.072 (2)	0.066 (3)	0.050 (2)	−0.046 (2)	0.0057 (17)	−0.0077 (18)
C22	0.0274 (14)	0.048 (2)	0.0346 (16)	−0.0111 (14)	0.0048 (12)	−0.0051 (14)
C23	0.0445 (16)	0.044 (2)	0.0421 (17)	−0.0176 (15)	0.0170 (13)	−0.0060 (14)
C24	0.0489 (18)	0.046 (2)	0.052 (2)	−0.0229 (16)	0.0177 (15)	−0.0197 (16)
C25	0.0345 (16)	0.071 (2)	0.0423 (18)	−0.0184 (16)	0.0033 (13)	−0.0147 (16)
C26	0.0498 (19)	0.043 (2)	0.055 (2)	−0.0075 (16)	−0.0040 (16)	−0.0145 (17)
C27	0.0478 (17)	0.0378 (19)	0.0358 (16)	−0.0148 (15)	0.0070 (14)	−0.0066 (13)
C28	0.060 (2)	0.041 (2)	0.056 (2)	−0.0209 (18)	−0.0010 (17)	−0.0017 (16)
C29	0.052 (2)	0.051 (2)	0.0461 (19)	−0.0036 (17)	0.0021 (17)	−0.0108 (16)
C30	0.0503 (18)	0.0351 (18)	0.0403 (17)	−0.0169 (15)	0.0087 (14)	−0.0068 (13)
C31	0.0526 (18)	0.049 (2)	0.0475 (19)	−0.0265 (16)	0.0005 (15)	−0.0109 (15)
C32	0.0440 (17)	0.053 (2)	0.0499 (19)	−0.0228 (16)	0.0066 (14)	−0.0128 (16)
C33	0.0501 (18)	0.0424 (19)	0.0410 (17)	−0.0242 (15)	0.0110 (14)	−0.0145 (14)
C34	0.0504 (18)	0.057 (2)	0.051 (2)	−0.0306 (17)	0.0056 (15)	−0.0148 (16)
C35	0.0447 (17)	0.052 (2)	0.0503 (19)	−0.0201 (16)	0.0106 (15)	−0.0124 (16)
C36	0.0428 (16)	0.0327 (17)	0.0388 (16)	−0.0159 (14)	0.0017 (13)	−0.0068 (13)
C37	0.0406 (16)	0.054 (2)	0.0496 (19)	−0.0217 (15)	−0.0027 (14)	−0.0165 (16)
C38	0.0336 (15)	0.047 (2)	0.056 (2)	−0.0125 (14)	0.0039 (14)	−0.0179 (16)
C39	0.0412 (16)	0.0313 (17)	0.0349 (15)	−0.0129 (13)	−0.0001 (13)	−0.0014 (12)
C40	0.0410 (16)	0.0396 (18)	0.0363 (16)	−0.0190 (14)	−0.0026 (13)	−0.0017 (13)
C41	0.0373 (15)	0.0380 (18)	0.0424 (17)	−0.0153 (14)	0.0019 (13)	−0.0034 (14)
C42	0.0453 (18)	0.0394 (19)	0.0408 (17)	−0.0168 (15)	−0.0003 (14)	−0.0064 (14)
N1	0.0461 (15)	0.0501 (18)	0.0486 (16)	−0.0171 (13)	0.0141 (12)	−0.0197 (14)
N2	0.0394 (13)	0.0397 (17)	0.0318 (13)	−0.0066 (11)	0.0055 (11)	−0.0073 (11)
N3	0.0463 (14)	0.0432 (17)	0.0330 (13)	−0.0152 (13)	0.0063 (11)	−0.0126 (12)
N4	0.0558 (17)	0.0525 (19)	0.0431 (16)	−0.0188 (14)	0.0153 (12)	−0.0131 (14)
N5	0.0540 (17)	0.061 (2)	0.0483 (17)	−0.0254 (15)	0.0162 (13)	−0.0189 (15)
N6	0.0418 (14)	0.0571 (19)	0.0367 (14)	−0.0191 (13)	0.0087 (11)	−0.0156 (12)
N7	0.0356 (12)	0.0409 (16)	0.0318 (13)	−0.0140 (12)	0.0016 (10)	−0.0070 (11)
N8	0.0416 (14)	0.058 (2)	0.0449 (15)	−0.0192 (14)	0.0105 (12)	−0.0169 (14)
N9	0.0501 (15)	0.0552 (18)	0.0448 (15)	−0.0270 (14)	0.0108 (12)	−0.0181 (13)
N10	0.0485 (15)	0.0496 (17)	0.0436 (15)	−0.0228 (13)	0.0087 (12)	−0.0168 (12)
O1	0.0503 (14)	0.0697 (18)	0.0605 (15)	0.0017 (13)	−0.0033 (12)	−0.0273 (13)
O2	0.0551 (13)	0.0705 (17)	0.0395 (12)	−0.0129 (12)	−0.0039 (11)	−0.0142 (11)
O3	0.0803 (18)	0.098 (2)	0.0607 (16)	−0.0336 (17)	0.0136 (14)	−0.0435 (15)
O4	0.0660 (18)	0.172 (4)	0.079 (2)	−0.037 (2)	0.0278 (15)	−0.069 (2)
Ag1	0.0708 (2)	0.1030 (3)	0.05629 (19)	−0.04152 (19)	0.03180 (14)	−0.03127 (17)
Ag2	0.06397 (19)	0.0992 (3)	0.05733 (19)	−0.04227 (18)	0.03016 (14)	−0.03882 (16)
O1W	0.162 (3)	0.130 (3)	0.0544 (17)	−0.101 (3)	0.0134 (18)	−0.0196 (17)
O2W	0.077 (2)	0.135 (3)	0.101 (2)	−0.0014 (19)	−0.0064 (18)	−0.070 (2)

### Geometric parameters (Å, °)

**Table:**

C1—C2	1.339 (5)	C22—C25	1.510 (4)
C1—N1	1.364 (4)	C23—C24	1.378 (4)
C1—H1	0.9300	C23—H23	0.9300
C2—N2	1.367 (4)	C24—H24	0.9300
C2—H2	0.9300	C25—N7	1.467 (4)
C3—N1	1.315 (4)	C25—H25A	0.9700
C3—N2	1.328 (4)	C25—H25B	0.9700
C3—H3	0.9300	C26—C28	1.338 (5)
C4—N2	1.463 (4)	C26—N8	1.366 (4)
C4—C5	1.499 (4)	C26—H26	0.9300
C4—H4A	0.9700	C27—N8	1.309 (4)
C4—H4B	0.9700	C27—N7	1.330 (4)
C5—C6	1.373 (5)	C27—H27	0.9300
C5—C10	1.385 (5)	C28—N7	1.363 (4)
C6—C7	1.382 (5)	C28—H28	0.9300
C6—H6	0.9300	C29—O3	1.232 (4)
C7—C8	1.377 (5)	C29—O4	1.241 (4)
C7—H7	0.9300	C29—C30	1.505 (4)
C8—C9	1.372 (5)	C30—C35	1.393 (4)
C8—C11	1.506 (4)	C30—C31	1.400 (4)
C9—C10	1.383 (4)	C31—C32	1.360 (4)
C9—H9	0.9300	C31—H31	0.9300
C10—H10	0.9300	C32—C33	1.403 (4)
C11—N3	1.470 (4)	C32—H32	0.9300
C11—H11A	0.9700	C33—C34	1.385 (4)
C11—H11B	0.9700	C33—N9	1.411 (4)
C12—N4	1.314 (4)	C34—C35	1.376 (4)
C12—N3	1.334 (4)	C34—H34	0.9300
C12—H12	0.9300	C35—H35	0.9300
C13—C14	1.338 (5)	C36—C37	1.387 (4)
C13—N3	1.359 (4)	C36—C41	1.403 (4)
C13—H13	0.9300	C36—N10	1.414 (4)
C14—N4	1.349 (4)	C37—C38	1.365 (4)
C14—H14	0.9300	C37—H37	0.9300
C15—C16	1.347 (5)	C38—C39	1.393 (4)
C15—N5	1.370 (5)	C38—H38	0.9300
C15—H15	0.9300	C39—C40	1.398 (4)
C16—N6	1.361 (4)	C39—C42	1.506 (4)
C16—H16	0.9300	C40—C41	1.349 (4)
C17—N5	1.313 (4)	C40—H40	0.9300
C17—N6	1.337 (4)	C41—H41	0.9300
C17—H17	0.9300	C42—O1	1.236 (4)
C18—N6	1.464 (4)	C42—O2	1.250 (3)
C18—C19	1.506 (4)	N1—Ag2	2.123 (3)
C18—H18A	0.9700	N4—Ag1	2.117 (3)
C18—H18B	0.9700	N5—Ag1	2.118 (3)
C19—C20	1.377 (5)	N8—Ag2^i^	2.132 (3)
C19—C24	1.386 (5)	N9—N10	1.249 (3)
C20—C21	1.381 (5)	Ag2—N8^ii^	2.132 (3)
C20—H20	0.9300	O1W—HW11	0.8487
C21—C22	1.377 (4)	O1W—HW12	0.8495
C21—H21	0.9300	O2W—HW21	0.8495
C22—C23	1.381 (4)	O2W—HW22	0.8489
			
C2—C1—N1	109.1 (3)	N7—C25—H25B	109.1
C2—C1—H1	125.4	C22—C25—H25B	109.1
N1—C1—H1	125.4	H25A—C25—H25B	107.8
C1—C2—N2	106.9 (3)	C28—C26—N8	108.8 (3)
C1—C2—H2	126.5	C28—C26—H26	125.6
N2—C2—H2	126.5	N8—C26—H26	125.6
N1—C3—N2	111.4 (3)	N8—C27—N7	111.3 (3)
N1—C3—H3	124.3	N8—C27—H27	124.4
N2—C3—H3	124.3	N7—C27—H27	124.4
N2—C4—C5	114.0 (2)	C26—C28—N7	107.0 (3)
N2—C4—H4A	108.8	C26—C28—H28	126.5
C5—C4—H4A	108.8	N7—C28—H28	126.5
N2—C4—H4B	108.8	O3—C29—O4	125.1 (3)
C5—C4—H4B	108.8	O3—C29—C30	117.9 (3)
H4A—C4—H4B	107.7	O4—C29—C30	117.0 (3)
C6—C5—C10	118.2 (3)	C35—C30—C31	118.0 (3)
C6—C5—C4	121.6 (3)	C35—C30—C29	121.9 (3)
C10—C5—C4	120.1 (3)	C31—C30—C29	120.1 (3)
C5—C6—C7	121.0 (3)	C32—C31—C30	121.5 (3)
C5—C6—H6	119.5	C32—C31—H31	119.3
C7—C6—H6	119.5	C30—C31—H31	119.3
C8—C7—C6	120.7 (3)	C31—C32—C33	120.1 (3)
C8—C7—H7	119.7	C31—C32—H32	119.9
C6—C7—H7	119.7	C33—C32—H32	119.9
C9—C8—C7	118.6 (3)	C34—C33—C32	118.9 (3)
C9—C8—C11	120.1 (3)	C34—C33—N9	116.5 (3)
C7—C8—C11	121.3 (3)	C32—C33—N9	124.6 (3)
C8—C9—C10	120.8 (3)	C35—C34—C33	120.7 (3)
C8—C9—H9	119.6	C35—C34—H34	119.7
C10—C9—H9	119.6	C33—C34—H34	119.7
C9—C10—C5	120.6 (3)	C34—C35—C30	120.8 (3)
C9—C10—H10	119.7	C34—C35—H35	119.6
C5—C10—H10	119.7	C30—C35—H35	119.6
N3—C11—C8	111.3 (2)	C37—C36—C41	119.0 (3)
N3—C11—H11A	109.4	C37—C36—N10	116.4 (2)
C8—C11—H11A	109.4	C41—C36—N10	124.6 (3)
N3—C11—H11B	109.4	C38—C37—C36	120.2 (3)
C8—C11—H11B	109.4	C38—C37—H37	119.9
H11A—C11—H11B	108.0	C36—C37—H37	119.9
N4—C12—N3	111.3 (3)	C37—C38—C39	121.6 (3)
N4—C12—H12	124.4	C37—C38—H38	119.2
N3—C12—H12	124.4	C39—C38—H38	119.2
C14—C13—N3	107.0 (3)	C38—C39—C40	117.1 (3)
C14—C13—H13	126.5	C38—C39—C42	122.0 (3)
N3—C13—H13	126.5	C40—C39—C42	120.9 (2)
C13—C14—N4	109.5 (3)	C41—C40—C39	122.3 (3)
C13—C14—H14	125.3	C41—C40—H40	118.9
N4—C14—H14	125.3	C39—C40—H40	118.9
C16—C15—N5	109.5 (3)	C40—C41—C36	119.8 (3)
C16—C15—H15	125.2	C40—C41—H41	120.1
N5—C15—H15	125.2	C36—C41—H41	120.1
C15—C16—N6	106.5 (3)	O1—C42—O2	126.0 (3)
C15—C16—H16	126.7	O1—C42—C39	116.6 (3)
N6—C16—H16	126.7	O2—C42—C39	117.2 (3)
N5—C17—N6	111.5 (3)	C3—N1—C1	105.9 (3)
N5—C17—H17	124.2	C3—N1—Ag2	124.4 (2)
N6—C17—H17	124.2	C1—N1—Ag2	127.9 (2)
N6—C18—C19	114.3 (3)	C3—N2—C2	106.7 (3)
N6—C18—H18A	108.7	C3—N2—C4	125.6 (3)
C19—C18—H18A	108.7	C2—N2—C4	127.3 (3)
N6—C18—H18B	108.7	C12—N3—C13	106.3 (3)
C19—C18—H18B	108.7	C12—N3—C11	126.6 (3)
H18A—C18—H18B	107.6	C13—N3—C11	127.1 (3)
C20—C19—C24	117.7 (3)	C12—N4—C14	105.9 (3)
C20—C19—C18	120.8 (3)	C12—N4—Ag1	126.4 (2)
C24—C19—C18	121.4 (3)	C14—N4—Ag1	127.3 (2)
C19—C20—C21	121.3 (3)	C17—N5—C15	105.4 (3)
C19—C20—H20	119.4	C17—N5—Ag1	130.1 (3)
C21—C20—H20	119.4	C15—N5—Ag1	124.5 (2)
C22—C21—C20	120.7 (3)	C17—N6—C16	107.0 (3)
C22—C21—H21	119.6	C17—N6—C18	125.5 (3)
C20—C21—H21	119.6	C16—N6—C18	126.9 (3)
C21—C22—C23	118.5 (3)	C27—N7—C28	106.8 (3)
C21—C22—C25	121.3 (3)	C27—N7—C25	125.2 (3)
C23—C22—C25	120.2 (3)	C28—N7—C25	128.0 (3)
C24—C23—C22	120.6 (3)	C27—N8—C26	106.1 (3)
C24—C23—H23	119.7	C27—N8—Ag2^i^	126.6 (2)
C22—C23—H23	119.7	C26—N8—Ag2^i^	125.8 (2)
C23—C24—C19	121.2 (3)	N10—N9—C33	114.3 (2)
C23—C24—H24	119.4	N9—N10—C36	114.8 (2)
C19—C24—H24	119.4	N4—Ag1—N5	170.34 (10)
N7—C25—C22	112.5 (2)	N1—Ag2—N8^ii^	160.25 (10)
N7—C25—H25A	109.1	HW11—O1W—HW12	105.1
C22—C25—H25A	109.1	HW21—O2W—HW22	104.9

Symmetry codes: (i) *x*+2, *y*−1, *z*−1; (ii) *x*−2, *y*+1, *z*+1.

### Hydrogen-bond geometry (Å, °)

**Table:**

*D*—H···*A*	*D*—H	H···*A*	*D*···*A*	*D*—H···*A*
O1W—HW12···O2W^iii^	0.85	2.11	2.879 (5)	151
O1W—HW11···O3^iii^	0.85	2.02	2.828 (4)	159
O2W—HW21···O4	0.85	1.93	2.758 (4)	163
O2W—HW22···O4^iv^	0.85	2.18	2.909 (6)	144

Symmetry codes: (iii) *x*, *y*+1, *z*; (iv) −*x*+3, −*y*−1, −*z*.
